# Molecular Mechanisms of Cardiotoxicity Induced by ErbB Receptor Inhibitor Cancer Therapeutics

**DOI:** 10.3390/ijms131012268

**Published:** 2012-09-26

**Authors:** Anne-Sophie Hervent, Gilles W. De Keulenaer

**Affiliations:** Laboratory of Physiopharmacology (Building T2), University of Antwerp, Universiteitsplein 1, 2610 Antwerp, Belgium; E-Mail: anne-sophie.hervent@ua.ac.be

**Keywords:** ErbB inhibitors, cancer, cardiotoxicity, heart failure

## Abstract

The introduction of the so-called “targeted therapies”, particularly those drugs that inhibit the activity of tyrosine kinases, has represented a remarkable progress in the treatment of cancer. Although these drugs improve survival rates in cancer, significant cardiotoxicity, manifesting as left vertricular dysfunction and/or heart failure, has emerged. The ErbB receptor tyrosine kinases are being pursued as therapeutic targets because of their important roles in normal physiology and in cancer. Besides the fact that the ErbB receptors are indispensable during development and in normal adult physiology, epidermal growth factor (EGFR) and ErbB2 in particular have been implicated in the development of many human cancers. This review focuses on the rationale for targeting members of ErbB receptor family and numerous agents that are in use for inhibiting the pathway. We summarize the current knowledge on the physiological role of ErbB signaling in the ventricle and on structural aspects of ErbB receptor activation in cancer and cardiac cells. We examine the underlying mechanisms that result in on-target or off-target cardiotoxicities of ErbB inhibitors, which can influence the design of future anticancer therapies.

## 1. Introduction

Cancer therapy has made remarkable progress with the development of “targeted therapeutics”. Whereas anthracyclines or radiotherapy are directed at all rapidly dividing cells, these therapies halt cancer cell proliferation and metastases with specific cytotoxicity. This targeted approach, mainly via inhibition of tyrosine kinase activity, has been proved to significantly reduce cancer progression and mortality (including gliomas [1], breast [2], ovarian [3], prostate [4], pancreatic [5], colorectal [6], lung and head and neck sqaumous cell carcinoma [7]).

The ErbB/HER family of receptor tyrosine kinases consists of four different proteins called EGFR/ErbB1/HER1, ErbB2/Neu/HER2, ErbB3/HER3, and ErbB4/HER4. Under normal physiological conditions, the ErbB receptors play crucial roles in propagating signals regulating cell proliferation, differentiation, motility and apoptosis [8]. Signal transduction pathways are initiated upon ligand-induced receptor homo- or heterodimerization and activation of tyrosine kinase activity. ErbB signaling is best known for its indispensable role during cardiac and neuronal development. It also has been implicated in the development of schizophrenia and several human cancers [9,10]. As overexpression of EGFR and ErbB2 receptors is often found in several human tumours such as breast, lung, head, and neck [11], ErbB receptors have been intensely pursued as therapeutic targets [8]. There are two general classes of ErbB-targeted therapeutics: humanized monoclonal antibodies (mAbs) directed against receptor tyrosine kinases and small-molecule tyrosine-kinase inhibitors (TKIs), targeting both receptor and nonreceptor tyrosine kinases. Although many of these therapies are either in clinical use or in clinical development, several studies have revealed unanticipated side effects, including cardiotoxicities, ranging from asymptomatic LV dysfunction to symptomatic congestive heart failure (CHF) [12]. Not all TKIs exert the same toxicity on the heart muscle, indicating that this is not a TKI “class effect”. Toxicity needs to be determined for each agent on a case-by-case basis. Following Force *et al*., tyrosine-kinase-targeted therapies can, therefore, be further subdivided in treatments with known (or likely) and low cardiotoxic risk (Table 1) [13].

It is essential to emphasize that not the level of expression, but rather the function of certain tyrosine kinases in the cardiomyocyte correlate with the toxicity induced by their corresponding inhibitors. In addition, rates of cardiotoxicity associated with TKIs are generally underestimated. The latter has several reasons. First, clinical trials have not included predefined cardiac endpoints. The identification of cardiotoxicity has, as a consequence, largely been based on medical history and physical examination [14]. Second, the diagnosis of CHF in patients with cancer can be difficult. They have many reasons other than LV dysfunction to develop the cardinal symptoms of CHF (dyspnoea, fatigue and oedema). Finally, by excluding older patients with existing comorbidities (cardiovascular disease) and due to the shorter duration of clinical trials, rates of heart failure, determined before Food and Drug Administration (FDA) approval, could underestimate rates that will be detected after approval.

The identification of the cardiotoxic mechanisms of ErbB-antagonists could help to guide future drug development. It could influence the development of new anticancer therapies in an attempt to achieve an effective treatment of the cancer while minimizing cardiotoxicity.

This review focuses on the rationale for targeting members of ErbB receptor family, in particular ErbB1 and ErbB2. We will review the biology of ErbB signaling in cancer and cardiac cells, the emerging opportunities to target this system with therapeutics and we will summarize what is known of the working mechanisms of currently FDA appoved ErbB targeted drugs. We will focus on the underling mechanisms of these targeted therapeutics that lead to cardiotoxicity and therefore discuss the two types of toxicity (“on- and off-target” toxicity), eludicating these mechanisms.

## 2. ErbB Receptors and Their Ligand

Subclass I of the receptor tyrosine kinase (RTK) superfamily consists of the ErbB receptors and comprises four members: ErbB1 (also called epidermal growth factor receptor (EGFR)), ErbB2, ErbB3 and ErbB4. All members have an extracellular ligand-binding region, a single membrane-spanning region and a cytoplasmic tyrosine-kinase-containing domain. The ErbB receptors are expressed in various tissues of epithelial, mesenchymal and neuronal origin. Under normal physiological conditions, activation of the ErbB receptors is controlled by the spatial and temporal expression of their ligands, which are members of the EGF family of growth factors [15,16].

**Figure 1:**
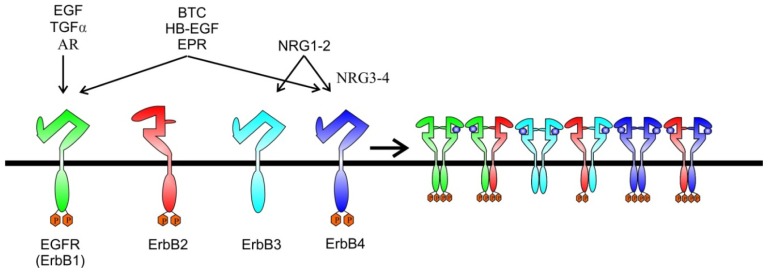
ErbB receptor tyrosine kinases and their ligands ( 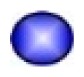). All four members of the ErbB receptor family share high homology in the extracellular domain and the kinase domain. However, ErbB3 lacks tyrosine kinase activity. So far no ligand has been found for ErbB2, which has been found to be the preferred dimerization partner for other receptors. EGFR (ErbB1), ErbB2, ErbB3 and ErbB4 have different ligand binding and signaling. EGF, epidermal growth factor; TGF α, transforming growth factor alpha; AR, amphiregulin; EPR, epiregulin; BTC, betacellulin; HB-EGF, heparin binding EGF; NRG, neuregulin. Ligand binding causes homo/heterodimerization by ErbB family members enhancing complexity of signal transduction.

**Figure 2:**
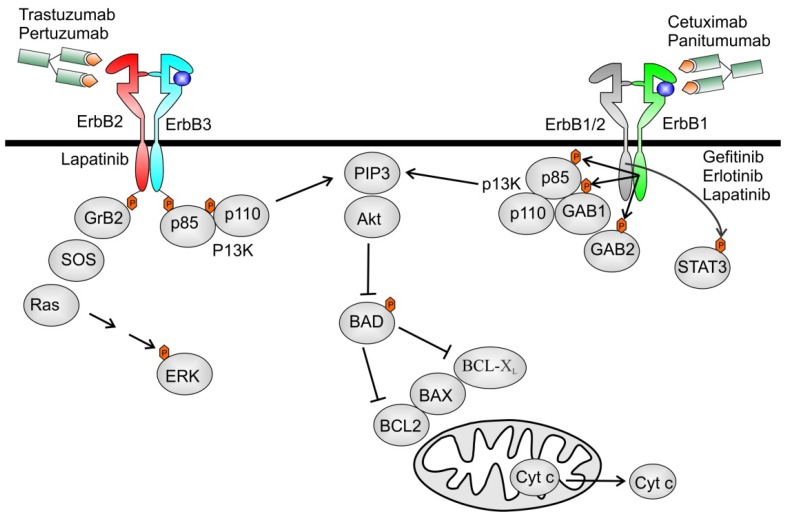
Mechanisms of ErbB signaling and inhibition in tumour cell. Oncogenic signaling incancer cells can be mediated by overexpressed ErbB2, ErbB2 homodimers or ErbB2/ErbB3 heterodimers, and also by ErbB1 homodimers or ErbB1/ErbB2 heterodimers. These events trigger signaling through a complex array of intracellular pathways that initiate and control a range of cellular processes. Out of many signaling event in a network, three key signaling pathways are the Ras-Erk (mitogen-activated protein kinase (MAPK)), the phosphatidylinositol 3-kinase (PI3K)–AKT and the Janus Kinase (JAK-STAT) pathway. The monoclonal antibodies (mAbs) cetuximab, panitumumab, trastuzumab and pertuzumab act extracellulary by blocking ligand binding and/or receptor dimerization. The small molecule tyrosine kinase inhibitors (TKI) gefitinib, erlotinib and lapatinib compete with ATP in the tyrosine-kinase domain of the receptor and block tyrosine kinase activity. Both interrupt downstream signaling. In addition to inhibiting ErbB signaling, mAbs might also mediate anti-tumour effects through antibody-dependent cell-mediated cytotoxicity (ADCC). Inhibition of ErbB2 by trastuzumab impairs all downstream events, in particular reversing BCL2-antagonist of cell death (BAD) inhibition, leading to a decrease in anti-apoptotic Bcl-xL expression. This leads to a destabilization of the mitochrondrial membrane and ATP depletion, cytochroom c release and caspase activation. Only signaling through these three pathways and known subcellular processes are shown here for simplicity.

The EGF family of ligands can be divided into three groups: the first includes EGF, transforming growth factor-α and amphiregulin, which bind specifically to EGFR/ErbB1; and the second includes betacellulin, heparin-binding EGF and epiregulin, which show dual specificity, binding both EGFR/ErbB1 and ErbB4. The third group is composed of the neuregulins (NRGs) and forms two subgroups based on their capacity to bind ErbB3 and ErbB4 (NRG-1 and NRG-2) or only ErbB4 (NRG-3 and NRG-4) (Figure 1) [17]. None of the ligands bind to ErbB2, but ErbB2 is the preferred dimerization partner for all the other ErbB receptors. ErbB3 has impaired kinase activity and only acquires signaling potential when it is dimerized with another ErbB receptor, such as ErbB2. Ligand binding to ErbB receptors induces the formation of receptor homo- and heterodimers and the activation of the intrinsic kinase domain, resulting in phosphorylation of specific tyrosine residues within the cytoplasmic tail. These phosphorylated residues serve as docking sites for intracellular signaling molecules. The ligand determines the tyrosine residues that are phosphorylated and hence the signaling molecules recruited. Three main pathways that can be stimulated upon activation of ErbBs are the mitogen-activated protein kinase (MAPK), the phosphatidylinositol 3-kinase (PI3K)–AKT and the Janus Kinase (JAK-STAT) pathway, all responsible for the regulation of cellular metabolism, growth and survival (Figure 2) [10,16,18,19].

The importance of ErbB receptors during development and in normal adult physiology is evident from analyses of genetically modified mice. Studies in mutant mice revealed that gene deletion of ErbB2 [20] and ErbB4 [21] or their ligand NRG-1 [22] leads to embryonic lethality caused by abnormal ventricular trabeculation. ErbB3-null mice display a different phenotype and die because of defective cardiac cushion formation [23] while deletion of ErbB1 leads to embryonic or early postnatal lethality which appears not to be primarily of cardiac origin [24,25]. Cardiac expression of ErbB ligands and receptors was shown to persist into adulthood, suggesting a role in postnatal cardiac physiology [26].

## 3. ErbB Receptors and Cancer

In many different cancer cell types, the ErbB pathway becomes hyperactivated by a range of mechanisms, including overproduction of ligands (e.g., TGF-α and NRG-1), overproduction of receptors, or constitutive activation of receptors. Both overexpression and structural alterations of EGFR/ErbB1 are frequent in human malignancies. Furthermore, in many tumours EGF-related growth factors are produced either by the tumour cells themselves or are available from surrounding stromal cells, leading to constitutive EGFR activation [27]. Gene amplification leading to EGFR overexpression is found in several human cancers, most commonly in brain tumours [28,29]. Overexpression is associated with higher tumour grade, higher proliferation and reduced survival. In gliomas, EGFR amplification is often accompanied by structural rearrangements that cause in-frame deletions in the extracellular domain of the receptor (EGFR vIII) [30]. Identical alterations have also been identified in carcinomas of the breast, lung and ovaries, suggesting broader implications for human cancer [31].

Of the four members of the family, ErbB2 is the most closely associated with carcinogenesis. *In vivo* studies using transgenic mice have demonstrated that overexpression of this receptor is able to induce mammary gland transformation, tumourigenicity and metastases formation, both ligand dependent and independent [32,33]. In humans, ErbB2 is found to be overexpressed in 20%–30% of invasive breast carcinomas due to gene amplification [34]. ErbB2 overexpression is also significant in ovarian, gastric and bladder cancer [35]. Furthermore, mutations in the kinase domain of ErbB2 have been identified in a small number of non-small-cell lung cancers (NSCLC) [36]. The catalytically inactive member of the ErbB family, ErbB3, is expressed in several cancers, but there is no evidence for gene amplication and overexpression is limited. However, several studies have established that the ErbB2/ErbB3 heterodimer functions as an oncogenic unit in ErbB2 amplified tumour cells [37].

The role of ErbB4 in oncogenic signaling is more controversial. Some studies have observed lower expression of ErbB4 in breast and prostate tumours relative to normal tissues, and an association with a relatively differentiated histological phenotype [38], but in contrast, childhood medulloblastomas often express ErbB4, whose co-expression with ErbB2 has a prognostic value [39].

## 4. ErbB Receptors as Targets for Cancer Therapy

Due to the central role of the ErbB system in the development of carcinomas, selective inhibition of aberrant tyrosine kinase activity has become an exciting focus of anticancer therapy. Most effort have concentrated on ErbB1 and ErbB2 owing to their increased expression in certain tumour cells relative to normal cells.

Two important types of ErbB inhibitors are in clinical use: humanized antibodies (mAbs) directed against the extracellular domain of EGFR or ErbB2 and small-molecule tyrosine-kinase inhibitors (TKIs) that compete with ATP in the tyrosine-kinase domain of the receptor. Therapeutic monoclonal antibodies (mAbs) bind to the ectodomain of the RTK with high specificity and thereby inhibits its downstream signaling by triggering receptor internalization and hindering ligand–receptor interaction. Unlike small-molecule inhibitors, mAbs also activate Fc-receptor-dependent phagocytosis or cytolysis by immune-effector cells such as neutrophils, macrophages and natural killer (NK) cells by inducing complement-dependent cytotoxicity (CDC) or antibody-dependent cellular cytotoxicity (ADCC) [40]. Small-molecule TKIs function as ATP analogues and inhibit EGFR signaling by competing with ATP binding within the catalytic kinase domain of RTKs. As a result, the activation of various downstream signaling pathways is blocked [41]. Therapeutic mAbs are large proteins (around 150 kDa) and are generally intravenously administered, whereas TKIs are orally available, synthetic chemicals (approximately 500 Da). Because of their inability to pass through the cellular membrane, mAbs can only act on molecules that are expressed on the cell surface or secreted [42] while small-molecule inhibitors can pass into the cytoplasm, and can therefore be developed to target any molecules regardless of their cellular location [43]. Typically, the advantage of therapeutic mAbs in cancer treatment is thought to depend on their capability to bind antigens expressed on the tumour-cell surface with a highly specific selectivity. Overall, TKIs are inherently less selective than mAbs and typically inhibit several kinases, some known and others not [44].

## 5. Cardiotoxicity

The goal of targeted therapy is a high efficacy with minimal side effects. Targeted therapies have been proven to significantly reduce cancer progression and mortality, but unfortunately, a major down-side effect involving the heart emerged in clinical trials [45]. This often occurs because pathways that drive tumourigenesis may also regulate survival of cardiomyocytes. Targeting these pathways in tumour cells may inherently lead to “on-target” toxicity, manifest as cardiomyopathy, because of inhibition of the same prosurvival kinases in normal cardiomyocytes.

The two types of toxicity will be explained to eludicate the underlying molecular mechanisms of TKI-derived cardiotoxicity. The first is “on-target” toxicity, wherein the tyrosine kinase target regulating cancer cell survival and/or proliferation also serves an import role in normale cardiomyocyte survival. Thus, inhibition leads to adverse consequences in the heart. Second, “off-target” occurs when a TKI leads to toxicity via inhibition of a kinase or pathway not intended to be a target of the drug. We will summarize examples of on-target toxicity of ErbB inhibitors [46].

### 5.1. Agents Targeting ErbB1

EGFR is overexpressed in several cancers, including colon, breast and brain, and diverse drugs targeting ErbB1 have been developed. Anti-ErbB1 mAbs and TKIs target distinct domains of EGFR, the extracellular ligand-binding domain and intracellular tyrosine kinase domain of the receptor, respectively. Cetuximab (also known as C225; Erbitux^®^) is a chimeric IgG1-isotype mAb that binds to ErbB1 with high affinity and abrogates ligand-induced ErbB1 phosporylation [47,48]. Structural analysis by Li *et al*. showed that the interaction of the mAb cetuximab with ErbB1 results in the partial occlusion of the ligand-binding region (L2) and steric hindrance preventing the receptor from adopting the extrended conformation required for dimerization [49]. Cetuximab is approved for treatment of metastatic colorectal cancers expressing ErbB1, for locally advanced squamous cell carcinoma of the head and neck and in non-small-cell lung cancer (NSCLC). In addition, panitumumab (Vectibix^®^) is developed as a fully human IgG2-isotype mAb against ErbB1 and is approved for the treatment of patients with EGFR-expressing metastatic colorectal cancers. By contrast, small-molecule TKIs gefitinib and erlotinib specifically inhibit EGFR phosphoryation and downstream signaling pathways. Gefitinib (Iressa^®^) and erlotinib (Tarceva^®^) are both efficacious against ErbB1-expressing cancers such as NSCLC and gliomas [50,51]. Erlotinib in combination with an anti-metabolite, gemcitabine, is also approved for treating advanced pancreatic cancer [52]. Although small-molecule inhibitors are generally thought to be less specific than therapeutic mAbs, this lower specificity is potentially advantageous, yet with some risk of increased toxicity. They have the ability to inhibit several signaling pathways at clinically possible plasma concentrations [53]. In particular, small-molecule EGFR TKIs show varying degrees of cross-reactivity for the ErbB family members, which might account for their potent anti-tumour effects when used in combination with a more selective mAb against ErbB1. Huang *et al*. demonstrated more profound tumour regression and regrowth delay in human lung cancer xenograft mice treated with the combination of cetuximab and gefitinib or erlotinib [44]. These date suggest that tyrosine kinase inhibitors may further modulate intracellular signalling that is not fully blocked by extracellular anti-ErbB1 mAb treatment.

#### Cardiotoxicity of ErbB1 Inhibitors

The adverse effects associated with ErbB1 inhibitors are mild. The most common, is skin rash resulting from the effects of EGR inhibition, possible due to the expression of EGFRs in the epidermis. Another common adverse effect in patients treated with TKI, but not in patients treated with mAbs, is diarrhoea [54]. The only severe toxicity reported with any of these agents is gefitinib-related interstitial pneumonitis [55]. Agents targeting solely ErbB1 (erlotinib, gefitinib) seem to have a low incidence of cardiotoxicity [56]. Since ErbB2 often dimerizes with ErbB1 after binding with ligand-bound ErbB1, the cardiotoxic effects observed in ErbB1-targeted interventions may be an indirect manifestation of impaired ErbB2 signaling.

### 5.2. Agents Targeting ErbB2

**Trastuzumab** (Herceptin^®^) is a humanized monoclonal antibody that inhibits the receptor tyrosine kinase ErbB2, which is overexpressed in approximately 25% of human breast cancer. Trastuzumab was first approved by the FDA in 1998 for the treatment of advanced metastatic breast carcinoma with overexpression of ErbB2 [2,57]. Since 2006 its use has broadened to the treatment of early breast cancer with the addition of trastuzumab to standard regimens. The addition of trastuzumab to chemotherapy (anthracyclines) reduces breast cancer relaps by 50% and mortality by 33% [58,59]. Anti-ErbB2 agents are increasingly used in the adjuvant setting after local regional treatment for a primary tumour [58,60].

Experimental and clinical data demonstrate several mechanisms of action of trastuzumab [61,62]. The main mechanism being the prevention of ErbB2 receptor dimerization, which is required for receptor activation and signal transduction. ErbB2 has no known ligand, instead, it has a fixed conformation that resembles the ligand-activated state. This structural characteristic is the reason why uncontrolled gene amplification and ErbB2 overexpression leads to ligand-independent constitutive ErbB2-ErbB3 heterodimer formation, continuous stimulation of downstream signaling pathways and uncontrolled cellular proliferation. Recent studies have established that the ErbB2/ErbB3/PI3K complex forms the major oncogenic unit in ErbB2 amplified tumour cells, and that sebsequent activation of Akt is the major oncogenic mechanism [63]. Other possible mechanisms of action are downregulation of ErbB2 abundancy. The binding of trastuzumab with its epitope located on the domain IV of ErbB2 induces internalization of the receptor, thus reducing the relative cell surface expression. Trastuzumab is also able to inhibit shedding of extracellular domain [64]. When overexpressed, ErbB2 undergoes proteolytic cleavage resulting in the release of the extracelluar domain and in the production of a truncated membrane-bound fragment, called p95, which is the ErbB2 active form of the receptor [65]. The inhibition of ErbB2 activity results in blockage of the signal transduction cascade depending on this receptor, including the Ras/Raf MAPKs and PI3K/Akt pathways connected with proliferation, survival, motility and angiogenesis. Finally, another antiproliferative mechanism of trastuzumab is due to the activation of ADCC, which is mainly due to the activation of NK cells, expressing the Fc gamma receptor, which can be bound by the Fc domain of trastuzumab. This event activates the lysis of cancer cells bound to trastuzumab (Figure 2) [66,67].

**Lapatinib** (Tykerb^®^), a small molecule dual kinase inhibitor of ErbB2 and EGFR/ErbB1, has recently been introduced as an alternative to, or as an add-on therapy with, trastuzumab [68–70]. The orally available 4-anilinoquinazoline compound works by binding reversibly to the intracellular ATP-binding site of the kinase, thereby blocking phophorylation and activation of the receptor. In contrast to trastuzumab, lapatinib inhibits both constitutive and ligand-induced ErbB signaling [71]. Since lapatinib does not interact with the extracellular domain of ErbB2, it is able to inhibit trastuzumab resistant tumour cells that express p95 ErbB2. It is also able to traverse blood-brain barrier, thus accessing central nervous system metastasis [72,73].

**Figure 3:**
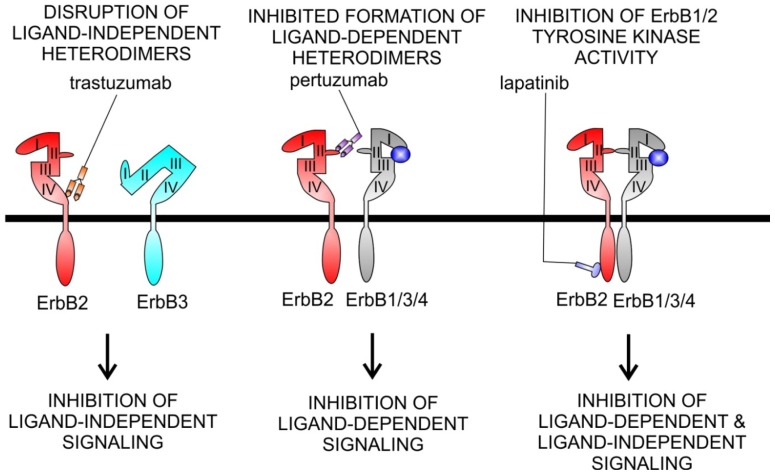
Mode action of current ErbB2 inhibitors. Trastuzumab, pertuzumab, and lapatinib have a different mode of action on ErbB signaling. Trastuzumab (**left**) is a humanized monoclonal antibody to subdomain IV of ErbB2. This leads to disruption of ErbB2-ErbB3 complexes, formed when ErbB2 is overexpressed, and in absence of ligand-binding to ErbB3. The mechanisms of this disruption are still unclear. Disruption of these complexes inhibits PI3K signaling and Akt activation and explains the antiproliferative effects of trastuzumab in ErbB2-amplified tumour cells. Effects of trastuzumab on ligand-induced dimerization of ErbB receptors seem minor or absent. Pertuzumab (**middle**) is a humanized monoclonal antibody to subdomain II, the dimerization arm of ErbB2. Pertuzumab leads to inhibition of ligand-induced ErbB2 signaling, not of ligand-independent ErbB2 signaling. Lapatinib (**right**) is a small molecule tyrosine kinase inhibitor of ErbB1 and ErbB2. Lapatinib blocks tyrosine kinase activity, independently of whether this activity has been triggered by a ligand or not.

**Pertuzumab** (Omnitarg^®^) is a humanized mAb directed against the extracellular domain of ErbB2 that blocks the ability of ErbB2 to heterodimerize with other members of the family. This impairs ErbB2/ErbB3 and ErbB2/ErbB1 heterodimers both in ErbB2 overexpressing and in cells that express normal levels of ErbB2 [74]. Pertuzumab was given as a single agent in phase II studies in ovarian, breast, prostate, and lung cancers [4,75–78]. Different from the mode of action of trastuzumab, pertuzumab functions by inhibiting the association of ErbB2 with ligand-induced ErbB members. As trastuzumab binds to domain IV of ErbB2, a region not involved in receptor dimerization, pertuzumumab binds ErbB2 near the centre of the domain II dimerization arm (Figure 3) [79,80]. This feature might partly explain why pertuzumab inhibits the growth of tumours that express low ErbB2 levels, whereas trastuzumab does not [74]. In addition, due to their complementary modes of action, therapeutic efficacy augments when pertuzumab and trastuzumab are given in combination [81,82].

#### Cardiotoxicity of ErbB2 Inhibitors

ErbB2 antagonists prolong survival in cancer, but also interfere with homeostatic processes in the heart. ErbB2 is the preferred coreceptor for ErbB4, which is activated by NRG-1. This epidermal growth factor (EGF)-like growth factor is released from endothelial cells in the endocardium and in the myocardial microcirculation, hence contributing to intercellular crosstalk in the ventricle. After the observation that gene deletion of NRG-1 or ErbB receptors resulted in cardiac malformation or dilated cardiomyopathy, cardiac cell and tissue responses to NRG-1 have been studied. NRG-1 was observed to increase the cardiomyocyte resistance to apoptotic cell death [26,83], to induce cardiomyocyte hypertrophy [26,83], mitotic growth [84] and cell elongation with improved cell-cell adhesion [85]. In addition, NRG-1 is involved in angiogenesis [86] and in the nitric oxide synthase-dependent densensitization of adrenergic stimulation [87]. Apart from inducing a “cardioprotective program” *in vitro*, Bersell *et al*. recently reported that NRG-1 injections in adult mice promote myocardial regeneration along with increased cardiomyocyte cell cycle activity, leading to improved functionality after myocardial infarction [84]. Based on the indispensable role of NRG-1/ErbB system in the heart and the analogy between ErbB2 knockout-induced cardiomyopathy and trastuzumab-induced heart failure, many have concluded that trastuzumab causes cardiotoxicity by blocking the physiological actions of ErbB2 in the heart [88]. Although reasonable and convincing, this hypothesis still needs further investigation.

The cardiac side effects of trastuzumab, manifesting as a decrease in left ventricular ejection fraction (LVEF) or as symptomatic CHF, was noticed in the initial trials. Herein, trastuzumab was administered on top of standard therapy, which consisted of either paclitaxel or doxorubicin and cyclophosphamide. The incidence of cardiotoxicity ranged from 1% to 4% with paclitaxel alone to 11% when trastuzumab was administered on top of paclitaxel. Anthracyclines, such as doxorubicin, are themselves cardiotoxic, but addition of trastuzumab leads to a synergistic increase in incidence of cardiac symptoms from 7% with trastuzumab alone, to 27% when anthracyclines were administered concurrently [89]. The mechanisms of trastuzumab-induced cardiotoxicity is not fully understood, but is distinct from that of anthracyclines. Anthracyclines cause type I cardiotoxicity which is dose-dependent, irreversible and normally associated with biopsy changes, whereas trastuzumab causes type II cardiotoxicity, which is dose-independent, largerly reversible and does not produce ultrastructural changes on histological examination [90]. While the molecular basis of type II cardiotoxicity is largely unknown, several mechanisms of anthracycline induced type I cardiotoxicity are described. Anthracycline-induced cardiomyopathy is strongly linked to an increase in cardiac oxidative stress, as evidenced by reactive oxygen species (ROS) induced damage such as lipid peroxidation, along with reduced levels of antioxidants and sulfhydryl groups. Myofibrillar deterioration and intracellular calcium dysregulation are also important mechanisms commonly associated with anthracyclin-induced cardiotoxicity. Not only are cardiomyocytes a target of anthracycline induced apoptosis, but endothelial cells are also affected, as indicated by endothelial caspase activation and internucleosomal DNA degradation [91–93].

Surprisingly, unlike trastuzumab, clinical trials suggest that lapatinib has minimal cardiac toxicity. Two published phase I trials involving 67 and 81 patients respectively with advanced refractory solid malignancies reported minimal cardiotoxicity [94], and in phase III trials both with and without trastuzumab pretreatment, lapatinib was associated with only a 2.5% incidence of asymptomatic decreased LVEF [94,95]. These low rates of observed cardiotoxicity may be explained by the “biased” design of these trials, including patients that have already supported trastuzumab therapy without cardiotoxic side effects, and by starting lapatinib treatment only late after anthracyclines [96]. Nevertheless, underlying mechanisms for the observed differences in cardiotoxicity between traztuzumab and lapatinib have been proposed. Unlike small-molecule inhibitors, mAbs can inititate ADCC and CDC that could augment cardiotoxicity [41]. Indeed, trastuzumab, which is an immunoglobulin G1 (IgG1), has been demonstrated to elicit ADCC against ErbB2^+^ tumour cells [97]. However, pertuzumab, another anti-ErbB2 IgG1 antibody, has shown a very low frequency of cardiac side effects in clinical trials [75,78,81,82,98], suggesting that ADCC might not explain the trastuzumab cardiotoxicity. Another possible explanation could include the interference of trastuzumab with the ErbB2/ErbB3 heterodimer, triggering an increased pro-apoptotic Bcl-xS expression, a concurrent decrease in anti-apoptotic Bcl-xL expression. This leads to a destabilization of the mitochrondrial membrane and ATP depletion, cytochroom c release and caspase activation, impairing the contractile function of the cardiomyocyte. Lapatinib might alter or abolish the effect of trastuzumab on Bcl-x activity, enabling to reduce trastuzumab cardiotoxicity when they are administered together [101]. Finally, cardioprotective AMPK activation could also explain the discrepancy in cardiotoxicity. While lapatinib promotes AMPK activity and increases ATP production, trastuzumab inhibits AMPK, leading to ATP depletion and loss of contractile function of cardiomyocytes [100].

### 5.3. Agents Targeting ErbB3/ErbB4

ErbB3 appears to play a central role in oncogenic signaling, where the ErbB2/ErbB3 heterodimer functions as an ongenic unit, the role of ErbB3 being to activate the PI3K/Akt pathway [37]. The role of ErbB4 in oncogenic signaling is more controversial, with some suggestion that it may inhibit cell proliferation. Currently there are no FDA approved inhibitors for ErbB3/4 in clinical use, although anti-ErbB3 mAbs are being developed [101,102]. Clinical use of pertuzumab has revealed a role for ErbB3, in that low levels of ErbB3 expression are associated with patient responses in ovarian cancer [103].

## 6. Concluding Remarks

The decrease in morbidity and mortality achieved with targeted therapies represents a milestone in cancer treatment. However, a growing number of cancer patients are becoming victims of their doctors’ success, experiencing cardiovascular side-effects of anti-cancer treatments, including the induction of heart failure. Risk of cardiotoxicity is underestimated in current clinical trials which generally lack older patients and patients with significant co-morbidities. Furthermore, the risk of adverse events may increase as the novel agents target the growth and survival pathways of the healthy cardiomyocyte (notably the PI3K/Akt pathway and the ERK1/2 cascade). The combined use with each other or the use with other cytotoxic chemotherapeutics is also not beneficial. Our increased understanding of the molecular and structural characteristics of the ErbB family has been essential for the rational development of ErbB-targeted inhibitors. Nevertheless, there are numerous details to be revealed concerning the molecular mechanism underlying anti-ErbB drug-related cardiotoxicity. Considering the role of the oncogenic ErbB2-ErbB3 unit, it will be interesting to determine if activating ErbB3 mutations are uncovered in tumours that have low ErbB2 expression. Another concern will be to investigate the role of inhibiting ErbB4 by multitargeted ErbB-inhibitors determining in this way whether targeting multiple ErbB receptors will lead to unacceptable toxicity. The risk for cardiovascular side effects must be carefully balanced against potential benefits of treatment. For cancers with very poor prognosis, short-term cardiac side effects that reduce quality of life are relevant. Delayed toxicity, on the other hand, fall to a lower priority. In cancers with high likelihood of long-term survival and in the neo-and adjuvant setting, it is very important to consider cardiovascular risks. Anyhow, to make sure that the cured cancer patient does not succumbs treatment’s side effects, we need to achieve effective cancer treatment, while minimizing cardiac toxicity.

Improving cardiovascular outcomes in patients undergoing cancer treatment requires appropriate risk assessment, monitoring, and long-term follow-up care. Baseline cardiovascular assessment, before starting cancer treatment, and cardiologic surveillance during and after its completion by the evaluation of LVEF using echocardiography, are essential for detecting cardiotoxicity. However, LVEF measurement is a relatively insensitive tool for detecting subclinical myocardial injury. A new approach, based on the use of cardiac biomarkers and tissue Doppler imaging, has emerged, proving to be a more sensitive and specific tool for the early identification, assessment, and monitoring of drug-induced cardiac injury. Cardiospecific biomarkers, such as serum troponin and brain natriuretic, show high diagnostic efficacy in the early subclinical phase of the disease before the clinical onset of cardiomyopathy. Finally, the oncologist and cardiologist should collaborate to improve disease prognosis and patient overall survival.

## Figures and Tables

**Table 1:** Cardiotoxicity of ErbB-targeted therapeutics.

Agent	Class	Target	Malignancies	Other toxicity
***Drugs with known or likely cardiotoxicity***				
Trastuzumab (Herceptin^®^)	Humanized mAb	ErbB2	ErbB2^+^ breast cancer	Infusion reactions, neutropaenia
***Drugs with low cardiotoxicity*** *				
Lapatinib (Tykerb^®^)	TKI	EGFR/ErbB1; ErbB2	ErbB2^+^ breast cancer, ovarian cancer, gliomas, NSCLC	Skin rash, diarrhoea
Cetuximab (Erbitux^®^)	Chimeric mAb	EGFR/ErbB1	CRC, squamous cell carcinoma of head/neck	Skin rash, infusion reactions, interstitial lung disease, hypmagnesaemia
Panitumumab (Vectibix^®^)	Human mAb	EGFR/ErbB1	CRC	Skin rash
Gefitinib (Iressa^®^)	TKI	EGFR/ErbB1	NSCLC, gliomas	Skin rash, nausea, diarrhoea, interstitial lung disease
Erlotinib (Tarceva^®^)	TKI	EGFR/ErbB1	NSCLC, pancreatic cancer, gliomas	Skin rash, nausea, diarrhoea, interstitial lung disease
Pertuzumab (Omnitarg^®^)	Humanized mAb	ErbB2	Breast, ovarian, prostate cancer, NSCLC	Skin rash

* Rate of cardiotoxicity is only known for trastuzumab. Therefore others represent best guesses. mAb, monoclonal antibody; TKI, tyrosine kinase inhibitor; EGFR, epidermal growth factor receptor; CRC, colorectal cancer; NSCLC, non-small-cell-lung cancer.
